# Association Between Sugar-Sweetened Beverage Consumption as Meal Substitutes, Workload, and Obesity in Nurses: A Cross-Sectional Study

**DOI:** 10.3390/ijerph16244984

**Published:** 2019-12-07

**Authors:** Ting-Ti Lin, Yue Leon Guo, Christopher Gordon, Elizabeth Cayanan, Yi-Chuan Chen, Chung-Mei Ouyang, Judith Shu-Chu Shiao

**Affiliations:** 1 School of Nursing, National Defense Medical Center, 161, Sec. 6, Minquan E Road., Neihu Dist., Taipei 11490, Taiwan; tlin@mail.ndmctsgh.edu.tw; 2 Environmental and Occupational Medicine, National Taiwan University (NTU) College of Medicine and NTU Hospital, 1, Sec. 1, Jen-Ai Road, Taipei 10051, Taiwan; leonguo@ntu.edu.tw; 3 Susan Wakil School of Nursing and Midwifery, The Faculty of Medicine and Health, University of Sydney, 88 Mallett St., Camperdown, NSW 2050, Australia; christopher.gordon@sydney.edu.au (C.G.); elizabeth.cayanan@sydney.edu.au (E.C.); 4 CIRUS Centre for Sleep and Chronobiology, Woolcock Institute of Medical Research, Glebe 2037, Australia; 5 School of Nursing, College of Medicine, National Taiwan University, 1, Sec. 1, Jen-Ai Rd., Taipei 10051, Taiwan; yichuan1201@gmail.com; 6 Department of Nutrition, National Taiwan University Hospital Hsin-Chu Branch, 25., Ln. 422, Sec. 1, Jingguo Rd., HsinChu City 33059, Taiwan; maggieoy@gmail.com

**Keywords:** eating behaviors, empty calorie beverage, nurse, occupational stress, weight gain

## Abstract

*Background*: High occupational stress has been associated with altered eating behaviors and obesity. Occupational stress is reported to be high in Asian countries. Furthermore, many Asian countries are increasingly consuming Western-type foods (e.g., incorporating drinks with meals) which collectively may also be contributing to obesity. Therefore, the aim of this study was to examine (a) associations between sugar-sweetened beverage (SSB) consumption as meal replacement and obesity and (b) associations between workload and substituting meals with SSB in nurses. *Methods*: A representative sample of 854 hospital-based nurses completed a structured questionnaire about SSB consumption, workload, and body mass index (BMI). Log binomial regression models were employed to test associations between SSBs and obesity rates and associations between workload and SSBs. *Results*: Most participants (57.6%) consumed SSBs as meal replacements during work. This was related to high workloads during shifts. Substituting SSBs for meals was significantly associated with increased likelihood of obesity (aPRR = 1.4, 95% CI (1.1, 1.7)). Workload was positively associated with SSB intake as meal substitutes (aPRR = 1.4, 95% CI (1.2, 1.6)). *Conclusions*: Our findings show that SSBs are used as meal substitutes and is due to the workload demands. Sugar-sweetened beverage consumption is also positively associated with the increased likelihood of obesity. Interventions that modify workloads and decrease SSB consumption may improve workers’ eating behaviors and health.

## 1. Introduction

Globally, it has been estimated that one-third of working populations is either overweight or obese (33%–36%) [1,2]. This has serious population-level consequences, as obesity has been shown to be an independent risk factor for chronic diseases (e.g., type 2 diabetes) [3,4,5,6,7]. While the causes of obesity are complex, numerous studies have shown that increased sugar-sweetened beverage (SSB) consumption can be obesogenic [6,8,9,10]. It has been documented that hospital nurses tend to cope with fatigue by consuming sugar-sweetened food or SSBs at work [11]. In Asian countries, studies have suggested that unhealthy eating behaviors (e.g., food consumption misaligned with dietary recommendations) are prevalent among hospital nurses [12,13,14]. However, to the best of our knowledge, it is still unclear whether SSB intake at work is related to workers’ obesity, and what factors account for workers’ high prevalence rate of unhealthy eating behaviors. 

Occupational stress modifies obesity prevalence and dietary consumption of low-nutrient food. Studies have shown occupational stress is associated with obesity [15,16], whereas others have not shown any relationship [17,18]. Recent evidence showed that low job control was significantly associated with workers’ increased risk of long-term weight gain. Among female workers, high work demands contributed to long-term weight gain [1]. 

The Transactional Model of Stress and Coping suggests that stress is associated with people’s health behaviors that are influenced by how they appraise and cope with their stress [19]. Studies have linked occupational stress to increased likelihood of abnormal eating behaviors (e.g., emotional eating) [20,21,22] and greater intake of low-nutrient food (e.g., sweetened snacks) [23,24,25,26]. Time availability for food ingestion has also been linked to people’s healthy eating behaviors [27,28,29] with time pressure contributing more to workers’ decisions on food consumption compared to appetite [30]. Workers appear to prefer fast food consumption due to the high work demands and lack of break times [31]. Therefore, high occupational workload may negatively impact workers’ time availability for eating at work and influence workers’ decisions on healthy food consumption. 

Prior research work has suggested that occupational stress and time availability at work influence workers’ eating behaviors [20,21,22,29,30,31]. This research has focused mainly on how stress or stressful events are related to unhealthy eating behaviors or intake of low-nutrient food, but it remains unclear to what extent occupational stressors (e.g., workload) may contribute to SSB consumption as meal substitutes during work. It has been reported that unhealthy eating behaviors are prevalent among Asian hospital nurses [12,13,14]. Therefore, the aim of this paper was to examine (a) the associations between substituting meals with SSBs and obesity prevalence and (b) the associations between workload and SSB consumption as substitutes at work. We also estimated the adjusted population attributable risk (aPAR) of workload to suggest the possible implications of this study. We hypothesized that higher workloads would be associated with an increased likelihood of SSB meal replacement when working. 

## 2. Material and Methods

### 2.1. Study Design and Sample

A national cross-sectional study of Taiwanese hospital nurses was undertaken. The inclusion criteria included: (a) nurses working fulltime (i.e., at least 30 hours per week on average) in accredited Taiwanese hospitals [32] and (b) aged 20–65 years old. The exclusion criteria included: (a) administrative work position (e.g., Director) and (b) working less than six months as a nurse in the hospital.

We employed a random sampling method with partial replacement to sample participants across Taiwan. Briefly, random sampling was undertaken by listing all accredited hospitals in Taiwan [32] and then applying a systematic cluster sampling method to select approximately 2%–8% of the hospitals from each geographical region (i.e., north, middle, south, east) of Taiwan. Excel’s RANDBETWEEN function was employed to generate a random number between 1 and 10 for each region, and every tenth hospital was selected. 

The number of recruited nurses in each hospital varied depending on the level (i.e., medical centers, regional hospitals, district hospitals, or psychiatric hospitals) and total nurse workforce. If a sampled hospital refused to participate or employed less than ten nurses, we alternatively invited the same-level accredited hospitals in the same region as substitutes. As a result, 10 medical centers, 16 regional hospitals, and 10 district hospitals were sampled. In Taiwan, most medical centers are large hospital complexes with over a thousand beds [33]. 

### 2.2. Data Collection

This study protocol was approved by the Institutional Review Boards (IRBs) at the National Taiwan University Hospital (No. 201612249RINC). Participant recruitment was conducted using the following sequence: after obtaining permission from collaborating hospital, we mailed the questionnaire to each hospital with instructions for questionnaire distribution and collection including an expected timeline for collecting questionnaires from participants and returning questionnaires to the research team. Participants who returned the questionnaire received a gift with an equivalent value of NTD 100 (USD $3.2) for questionnaire completion. If questionnaires were not returned after three weeks, we contacted the hospital once for follow-up. As a result of this process, between September 2017 and December 2017, a total of 1500 questionnaires were distributed, and 1292 questionnaires were returned (Figure S1). The overall response rate was 86.1%. 

We excluded questionnaires from nurses working in non-acute care departments (e.g., outpatient departments, hemodialysis rooms, or nursing homes) (*n* = 254), part-time nurses (*n* = 26), and incomplete questionnaires (i.e., any missing values) (*n* = 158). 

### 2.3. Measurement Tools

We employed a structured questionnaire covering the following information: participants’ characteristics (e.g., demographics), body mass index (BMI) derived from self-reported body heights (cm) and weights (kg), workload, work characteristics (e.g., hospital work units), and dietary consumption (e.g., SSBs, food). 

#### 2.3.1. Workload

Two items in the unmet basic physiological needs subscale (i.e., not having an intact 30 minute mealtime, having no time to fulfill personal needs such as drinking water or going to the bathroom) and one item in the work demand subscale (i.e., not having enough time to meet patients’ demands) in the Nurse Occupational Stressor Scale (NOSS) [34] were employed to assess participants’ workload. The NOSS has been tested among Taiwanese hospital nurses and shown to have good reliability (i.e., test–retest reliability: 0.92 for the NOSS scale; Cronbach’s α: 0.69 for unmet basic physiological needs subscale). Each NOSS item was assessed on a 5 point Likert scale (i.e., never, rare, sometimes, often, always). In this study, a response indicating “always” or “often” was considered as a high workload level. 

#### 2.3.2. SSB Consumption at Work

A 10 item list of food/beverages (i.e., “desserts, snacks, instant noodles, fast food, fried food, handmade drinks, sugar-sweetened beverages, microwaved products, other”) was employed to assess participants’ food/beverage consumption at work. Each participant was asked the following questions: (a) “Do you consume any of the following items while working (please check all that apply)?”; (b) if any items were checked, they were further asked whether they substituted the aforementioned items for their meals during work time. We mainly focused on participants’ different types of SSB consumption, consisting of either “commercial SSB” (soft drinks, energy drinks) or “handmade drinks” (bubble tea, milk tea with tapioca) as a meal replacement during work time. 

#### 2.3.3. Covariates

Covariates included demographics (i.e., age, sex, educational attainment, marital status), other health-related personal characteristics (i.e., health history, habitual sleep duration on work days), and work characteristics (i.e., work tenure, weekly working hours, primary shift schedule in the past three months, hospital work units, and hospital levels). 

### 2.4. Statistical Analysis

The software JMP 9.0 (SAS Institute Inc., Cary, NC, 1989–2019) was employed for data analysis. Participants’ characteristics were summarized using descriptive statistics. Associations between SSB consumption as a meal replacement when working and the likelihood of obesity, defined as BMI >= 27 kg/m^2^, were analyzed using bivariate and age/sex-adjusted log binomial regression models.

To examine associations between workload and nurses’ SSB consumption at work, we employed bivariate log binomial regression models to estimate crude associations. Based on the log likelihood ratio test (LRT), with the significance level at 0.05, we identified significant covariates for respective pairs of predictors and outcome measures (e.g., workload and SSB consumption). We then adjusted the same-set of covariates (i.e., age, sex, health history, habitual sleep duration on work days, and hospital levels) in their respective models. Multivariate log binomial regression models were employed for hypothesis testing, and based on these results, the adjusted population attributable risk (aPAR) of each significant predictor was estimated. 

## 3. Results

A total of 854 participants were included in the final analysis. As shown in Table 1, participants were at the mean age of 30.9 years old (SD = 7.1), mostly female (96.1%), with a bachelor’s degree or above (74.8%), non-obese (i.e., BMI < 27) (85.6%), and did not have any chronic diseases (82.6%). Participants had worked as a nurse for an average of 8.3 years (SD = 6.8). Almost two-thirds of all participants (64.2%) consistently experienced high workloads.

Table 2 shows participants’ SSB consumption by workload. Almost half of all participants consumed commercial SSBs as a meal replacement when they encountered high workloads (40.9%–47.2%). They also replaced meals with handmade drinks during high workload shifts (53.9%–58.3%). 

Participants who substituted meals with commercial SSBs had a 40% increase in the likelihood of obesity (aPRR = 1.4, 95% CI (1.1, 1.7)), adjusting for age and sex (Table 3). Further, there was a 1.2 (95% CI (1.0, 1.5)) increased risk of obesity for participants who consumed handmade drinks as meal substitutes.

### Workload and SSB Consumption

Adjusting for covariates, there was a statistically increased risk of replacing meals with SSBs when workload was high (Table 4). Participants exposed to any of the high workload conditions had a higher risk of consuming SSBs as meal replacements (aPRR = 1.4, 95% CI (1.2, 1.6)). The estimated aPAR revealed that approximately 20% of nurses substituting SSBs for meals during work could be prevented (PAR = 19.0, 95% CI (10.8, 26.1)) if the high workload condition was removed. 

## 4. Discussion

To the best of our knowledge, this is the first study to link nurse’s SSB consumption with workload and obesity. The main finding was that nurses who used SSBs as meal replacements during work were more likely to be obese. Further, workload was positively associated with an increased risk of replacing meals with SSBs. Therefore, workers with high workloads were not eating meals but used SSBs as meal substitutes which may contribute to weight gain and obesity despite the increased work demands. With many Asian countries increasingly including Western-type foods and beverages into their diets, the need for workplace and dietary education is warranted. It is suggested that if healthcare workplace employers could reduce high workload conditions, up to one in five unhealthy eating behaviors during work time could be prevented.

There is considerable evidence showing the positive associations between obesity [6,8,10,35] and weight gain risk [36,37] and SSB consumption. The exact causes of these associations are not clear, but research suggests that SSB consumption is not counterbalanced by a reduction in food intake [38], and consumption of SSBs may increase appetite, leading to excess daily energy consumption [39]. While the participants in this study used SSBs as meal replacements at work, they may also have consumed meals with increased energy outside of work; however, this was not explored in this study. Several prospective cohort studies have revealed the efficacy of reducing the intake of SSBs on the risk of long-term weight gain [9] and the prevalence of overweight or obesity [5]. This may also reduce the risk of chronic diseases (e.g., type 2 diabetes, cardiovascular diseases) [3,4,5,6,7].

Occupational stress and reduced time availability at work have been associated with workers’ unhealthy eating behaviors [11,40]. A study of health care workers showed that not having enough time for a meal break was the most common barrier for workers’ healthy eating [29]. Among overweight/obese workers, Leung and colleagues [41] came to the same conclusion that workers tended to consume more low-nutrient foods in reaction to stress and time constraints in the workplace. We found that limited time availability due to the fact of a high workload increased the likelihood of SSB consumption as meal replacements and workers are consuming energy-rich drinks to cope with a lack of available meal-time which is changing eating behavior and is potentially obesogenic. It is not clear from our study which occupational factors contribute to the limited time availability for meals. This could be attributed to inadequate staffing or acuity and complexity of patients’ conditions. It is likely that there were multiple factors causing this phenomenon. There needs to be longitudinal research to determine the effects of these modified workplace behaviors on workers’ diet and obesity.

### Strengths and Limitations

A large representative sample of Taiwanese hospital nurses was recruited to examine associations between workload and SSB consumption at work. The main strength of this study is that the random sampling method increased the generalizability of this study. However, it should be noted that we excluded responses from hospital registered nurses working in non-acute care departments (e.g., outpatient departments) and part-time nurses. Therefore, the result of this study may only be generalized to hospital nurses working in acute care departments (e.g., surgical wards) and full-time nurses. In addition, several limitations should not be ignored in this study. First, using the cross-sectional study design, the causal inferences of associations between high workload and SSB consumption as meal replacements at work still could not be determined. However, using the log binomial regression model helps to reduce the possibility of inflated point estimates from logistic regression models. Second, the results were based on self-reported measures, and it is possible that recall bias may threaten the internal validity of the findings. Using a food/beverage checklist for dietary assessments may have contributed to measurement error which may have resulted in misclassification and underestimation of the association.

## 5. Conclusions

This is the first study to show that high workload is strongly associated with an increased likelihood of substituting SSBs for meals during work, and this was more pronounced in obese workers. The potential effects of these behaviors may lead to increased obesity and obesity-related diseases. This is particularly relevant to Asian countries, where existing dietary patterns are increasingly being influenced by Western foods, and consumption of high energy foods and drinks is increasing. Asian nurses may be particularly vulnerable due to the high workloads, and this study clearly demonstrates that they are substituting meals with SSB consumption. Furthermore, it was significantly influencing those nurses who were obese. It is recommended that workplace conditions guarantee an adequate mealtime to reduce the probability of SSB meal replacement.

## Figures and Tables

**Table 1:** Participants’ characteristics (*N* = 854).

Variables	Mean (SD)	*n* (%)
Demographics		
Age (in years)	30.9 (7.1)	
Female		821 (96.1)
Education attainment		
Vocational school/associate degree		215 (25.2)
Bachelor’s degree or above		639 (74.8)
Marital status		
Married/cohabitation		286 (33.5)
Single/divorced/widowed/separated		568 (66.5)
Health-related personal characteristics		
BMI (kg/m^2^)	22.5 (4.2)	
Obesity ^1^		
No		731 (85.6)
Yes		123 (14.4)
Health history		
None		705 (82.6)
Having at least one chronic disease ^2^		149 (17.4)
Sleep duration on work days (in hours)	6.9 (1.3)	
Work characteristics		
Work tenure as a RN (in years)	8.3 (6.8)	
Weekly working hours	44.2 (7.1)	
<48		612 (71.7)
>=48		242 (28.3)
Primary shift schedule in the past 3 months		
Day shift		285 (33.4)
Evening shift		201 (23.5)
Night shift		147 (17.2)
Rotating shift		221 (25.9)
Hospital work units		
Acute care ward ^3^		444 (52.0)
Special care units ^4^		410 (48.0)
Hospital levels		
Medical center		185 (21.7)
Workload conditions		
Not having 30 minute mealtime		438 (51.3)
No time to fulfill personal needs		279 (32.7)
Not having enough time to meet patients’ demands		108 (12.6)
Any of the above workload conditions		548 (64.2)

Abbreviation: SD—standard deviation; BMI—body mass index; RN—registered nurse. ^1^ Obesity was defined as BMI>= 27kg/m^2^ based on the recommendations from the Health Promotion Administration, Ministry of Health and Welfare. The overall BMI for general population ages 19–64 is between 23.0 and 24.0 kg/m^2^ among females. The prevalence rate of obesity among the female population ages 19–30 and 31–44 are 14.6% and 19.0%, respectively. In contrast, the overall BMI for the male general population age 19–64 is between 24.5 and 25.0 kg/m^2^. The respective prevalence rates of obesity among male population ages 19–30 and 31–44 are 17.0% and 32.3%, respectively; ^2^ chronic diseases included hypertension, diabetes, cardiovascular disease, hyperlipidemia, hyperthyroidism, hypothyroidism, cancer, gastrointestinal diseases, and polycystic ovary syndrome (PCOS); ^3^ acute care wards included surgical, medical, pediatric, gynecological, orthopedic, and psychiatric wards; ^4^ special care units referred to emergency room, operating room, and intensive care units.

**Table 2:** Sugar sweetened beverage consumption as meal replacements at work by workload conditions (*N* = 854).

Variables	Overall	Not Having a 30 Minute Mealtime	No Time to Fulfill Personal Needs	Not Having Enough Time to Meet Patients’ Demands	Any High Workload Condition ^1^
		(*n* = 438)	(*n* = 279)	(*n* = 108)	(*n* = 548)
	*n* (%)	*n* (%)	*n* (%)	*n* (%)	*n* (%)
Commercial SSBs	293 (34.3)	183 (41.8)	114 (40.9)	51 (47.2)	218 (39.8)
Handmade drinks	420 (49.2)	236 (53.9)	159 (57.0)	63 (58.3)	292 (53.3)
Total SSB intake ^2^	492 (57.6)	288 (65.8)	183 (65.6)	76 (70.4)	349 (63.7)

SSB: sugar-sweetened beverage. ^1^ refers to any of the high workload conditions listed; ^2^ total SSB intake refers to either commercial SSB or handmade drink consumption.

**Table 3:** Associations between SSB consumption and nurses’ obesity (***N*** = 854).

Variables	Obesity (BMI >= 27)	
	PRR (95% CI)	aPRR^1^ (95% CI)
Commercial SSBs	1.3 (1.1, 1.6) ^**^	1.4 (1.1, 1.7) ^**^
Handmade drinks	1.1 (0.9, 1.4)	1.2 (1.0, 1.5)
Total SSB intake ^2^	1.1 (0.9, 1.3)	1.2 (1.0, 1.4)

SSB: sugar-sweetened beverage, PRR: prevalence rate ratio, CI: confidence interval, aPRR: adjusted prevalence rate ratio. ^1^ the estimates were adjusted for age and sex; ^2^ total SSB intake refers to either commercial SSB or handmade drink consumption; ** *p* < 0.01.

**Table 4:** Associations between workload and SSB consumption at work (*N* = 854).

	% Exposed (E|D) ^1^	PRR (95% CI) ^2^	aPRR (95% CI) ^3^	aPAR (95% CI) ^4^
Workload conditions				
Not having a 30 minute mealtime	58.5	1.4 (1.2, 1.6) ^***^	1.4 (1.2, 1.6) ^***^	
No time to fulfill personal needs	37.2	1.3 (1.1, 1.5) ^***^	1.2 (1.0, 1.4) ^*^	
Not having enough time to meet patients’ demands	15.5	1.4 (1.1, 1.7) ^**^	1.3 (1.1, 1.7) ^*^	
Any of the above workload conditions	70.9	1.4 (1.2, 1.6) ^***^	1.4 (1.2, 1.6) ^***^	19.0 (10.8, 26.1)

PRR: prevalence rate ratio, aPRR: adjusted prevalence rate ratio, aPAR: adjusted population attributable risks, SSB: sugar-sweetened beverage. ^1^ E|D: among those who consumed SSBs, the proportion of participants who were exposed to the independent variables; ^2^ respective aPRRs were estimated using the log binomial regression models; ^3^ each model adjusted for age, sex, health history, habitual sleep duration on workdays, and hospital level; ^4^ respective aPARs were calculated based on the following formula: P(E|D) × ((aPRR − 1)/aPRR); * *p* < 0.05, ** *p* < 0.01, *** *p* < 0.001.

## Supplementary Materials

The following are available online at https://www.mdpi.com/1660-4601/16/24/4984/s1, Figure S1: Process of data collection.
