# Supplementary figures and images for: Association Between Sugar-Sweetened Beverage Consumption as Meal Substitutes, Workload, and Obesity in Nurses: A Cross-Sectional Study
Source: Int J Environ Res Public Health. 2019 Dec 7;16(24):4984. doi: 10.3390/ijerph16244984 (PMC6949918; doi:10.3390/ijerph16244984)

## Supplementary Materials

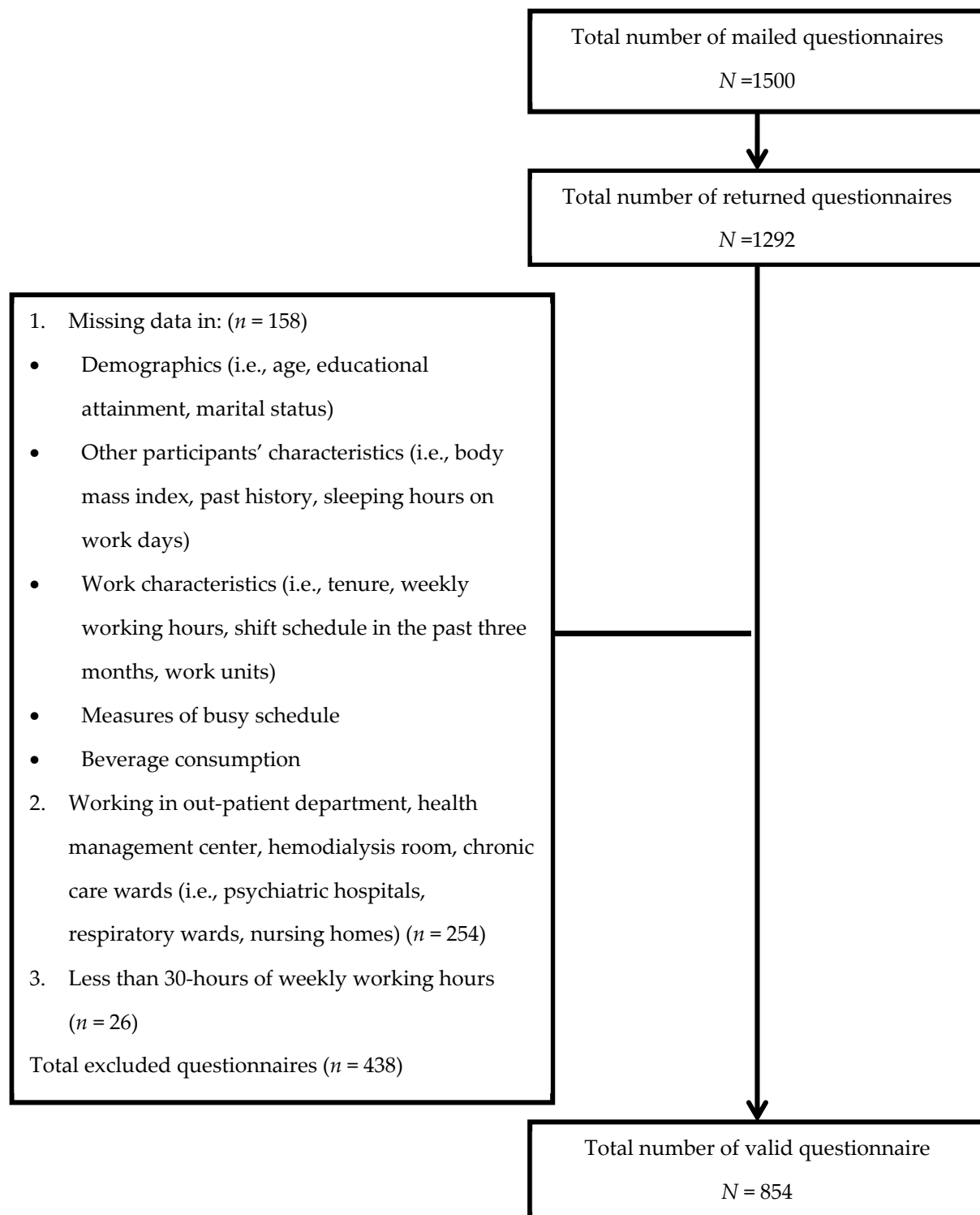

**Figure S1.** Process of data collection.

Supplement: Supplementary file 1 [file ijerph-16-04984-s001.pdf]
